# Cardiac-surgery associated acute kidney injury requiring renal replacement therapy. A Spanish retrospective case-cohort study

**DOI:** 10.1186/1471-2369-10-27

**Published:** 2009-09-22

**Authors:** Jose Ramon Perez-Valdivieso, Pablo Monedero, Marc Vives, Nuria Garcia-Fernandez, Maira Bes-Rastrollo

**Affiliations:** 1 Department of Anesthesia and Critical Care, Clinica Universidad de Navarra, University of Navarra, Pamplona, Spain; 2 Service of Nephrology, Clinica Universidad de Navarra, University of Navarra, Pamplona, Spain; 3 Department of Preventive Medicine and Public Health, University of Navarra, Pamplona, Spain

## Abstract

**Background:**

Acute kidney injury is among the most serious complications after cardiac surgery and is associated with an impaired outcome. Multiple factors may concur in the development of this disease. Moreover, severe renal failure requiring renal replacement therapy (RRT) presents a high mortality rate. Consequently, we studied a Spanish cohort of patients to assess the risk factors for RRT in cardiac surgery-associated acute kidney injury (CSA-AKI).

**Methods:**

A retrospective case-cohort study in 24 Spanish hospitals. All cases of RRT after cardiac surgery in 2007 were matched in a crude ratio of 1:4 consecutive patients based on age, sex, treated in the same year, at the same hospital and by the same group of surgeons.

**Results:**

We analyzed the data from 864 patients enrolled in 2007. In multivariate analysis, severe acute kidney injury requiring postoperative RRT was significantly associated with the following variables: lower glomerular filtration rates, less basal haemoglobin, lower left ventricular ejection fraction, diabetes, prior diuretic treatment, urgent surgery, longer aortic cross clamp times, intraoperative administration of aprotinin, and increased number of packed red blood cells (PRBC) transfused. When we conducted a propensity analysis using best-matched of 137 available pairs of patients, prior diuretic treatment, longer aortic cross clamp times and number of PRBC transfused were significantly associated with CSA-AKI.

Patients requiring RRT needed longer hospital stays, and suffered higher mortality rates.

**Conclusion:**

Cardiac-surgery associated acute kidney injury requiring RRT is associated with worse outcomes. For this reason, modifiable risk factors should be optimised and higher risk patients for acute kidney injury should be identified before undertaking cardiac surgery.

## Background

Cardiac surgery-associated acute kidney injury (CSA-AKI) requiring renal replacement therapy (RRT) increases mortality and hospital costs [1-3]. It accounts for approximately 4% of the patients [4]. A great number of the affected patients will remain RRT dependent after hospital discharge [5]. Research over the last years has identified some of the related factors, and has allowed doctors to classify patients according to their risk profile [1,6-10]. However, some of the proposed models have underestimated the risk of acute renal failure [11]. One step to further improve the accuracy of these scores is to clarify the influence of still unaccounted factors. After which, researchers might try to incorporate them into the equations. Besides, identifying high-risk patients will allow health care providers to give them more information and also to select them for more intensive therapies or new trials. Moreover, improving surgical planning will improve resource management and save costs.

We have conducted a retrospective case-matched cohort multicenter study to assess preoperative and perioperative variables and to try to identify the risk factors for acute kidney injury.

## Methods

After we received institutional research ethics board approval, we retrospectively reviewed a series of medical records from 1084 patients undergoing cardiac surgery in 24 Spanish hospitals. A request was made to collect data on all the patients who needed RRT after cardiac surgery in 2007 in every hospital, in addition to a variable number of consecutive patients based on age, sex, and treated before June 2007 at the same hospital by the same group of surgeons. Hospitals presented an overall mean rate for RRT after cardiac surgery in 2007 of 3.5% (1.0-6.5). Seven hospitals were not able to enroll all the control participants, due to a lack of compliance with the deadline. Patient demographics and preoperative risk factors, as well as intraoperative and postoperative data were gathered (Appendix). We excluded patients with preoperative RRT, off-pump surgery, or those patients who died within 48 hours after surgery. Finally, data from 998 patients undergoing coronary artery bypass grafting (CABG) surgery, valvular heart surgery, or both, were evaluated. Of those 998 patients, 134 were excluded for missing values (35 RRT cases and 99 non-RRT cases). The remaining 864 patients composed the study population. Among them, 174 patients who needed RRT after surgery could be matched to 690 controls.

We estimated the preoperative glomerular filtration rate (GFR) from serum creatinine using the Modification of Diet in Renal Disease (MDRD) Study equation [12].

Preoperative and intraoperative risk factors associated to CSA-AKI requiring RRT were assessed.

### Statistical analysis

Continuous variables are presented as means and standard deviations, and compared using Student's t-test. Categoric variables are shown as the percentage of the sample and compared with the Chi-squared test. Logistic regression analysis was performed to determine the factors independently associated with AKI requiring RRT and also to model the probability of mortality. Linear regression models were used to assess the association between AKI requiring RRT (exposure), and length of hospital stay (outcome). In both regression analyses we fitted a crude model (univariate, i.e., without any adjustment); an age, sex, and hospital source model; and a multivariate-adjusted model entering all variables with p < 0.1 in the bivariate analysis. Of the 864 patients in the entire study population, an additional propensity analysis using 137 best-matched pairs was also conducted. These matched pairs were well balanced for age, sex, body mass index, hospital source, previous glomerular filtration rates, and Euroscore using the nearest available matching on the estimated propensity score [13,14]

To assess the calibration and the discrimination of the models, we evaluated the Hosmer-Lemeshow goodness-of-fit statistic and the c-statistic, respectively.

Statistical analyses were conducted using SPSS 15.0 (SPSS Inc., Chicago, IL, USA).

## Results

Patient characteristics and clinical data are presented in Tables 1, 2, and 3.

**Table 1:** Preoperative characteristics of patients according to need of postoperative RRT.

	No postoperative RRT (n = 690)	Postoperative RRT (n = 174)	P value
Age in years	66.5 (10.6)	68.0 (10.2)	0.09

Men (%)	60.3	59.2	0.79

BMI in kg/m^2^	28.2 (4.4)	28.3 (4.2)	0.83

MDRD GFR in mL/min/1.73 m^2^	78.6 (26.4)	58.7 (27.7)	< 0.001

Basal Hemoglobin in g/dL	12.8 (1.8)	11.6 (1.8)	< 0.001

NSAIDs treatment (%)	14.6	10.9	0.21

ACE inhibitor treatment (%)	52.5	50.0	0.56

Diuretic treatment (%)	48.8	79.9	< 0.001

Statins treatment (%)	52.6	45.4	0.09

Diabetic patients (%)	29.9	42.0	0.002

Extracardiac arteriopathy (%)	13.5	18.4	0.1

COPD (%)	12.2	25.9	< 0.001

Hypertension (%)	65.4	67.2	0.64

Neurological dysfunction (%)	2.6	6.9	0.006

Congestive heart failure (%)	31.7	57.5	< 0.001

% Left ventricular ejection fraction	57.9 (11.3)	53.4(13.4)	< 0.001

NYHA class 4 (%)	7.7	21.8	< 0.001

Cardiogenic shock (%)	1.2	11.5	< 0.001

Infectious endocarditis (%)	2.3	13.8	< 0.001

Pulmonary hypertension (%)	13.0	33.3	< 0.001

Unstable angina (%)	14.5	14.4	0.97

Recent myocardial infarction (%)			0.23

No	88.8	89.1	

< 3 weeks	7.1	9.2	

> 3 and < 9 weeks	4.1	1.7	

Intra-aortic ballon pump (%)	1.4	7.5	< 0.001

Critical state (%)	2.8	18.4	< 0.001

Prior cardiac surgery (%)	8.4	25.9	< 0.001

Euroscore	5.91 (2.97)	9.24 (4.06)	< 0.001

Continuous variables are shown as mean (SD) and compared via Student's t-test. Categorical variables are shown as percentages and compared using Chi-squared test. BMI: Body mass index; MDRD: Modification of diet in renal disease; GFR: glomerular filtration rate; NSAID: Non-steroidal anti-inflammatory drug; ACE: Angiotensin-converting enzyme; COPD: Chronic obstrutive pulmonary disease; NYHA: New York heart association functional classification.

**Table 2:** Intraoperative characteristics of patients according to need of postoperative RRT.

	No postoperative RRT (n = 690)	Postoperative RRT (n = 174)	P value
Urgent surgery (%)	4.2	22.4	< 0.001

Surgery type (%)			< 0.001

CABG only	30.3	13.8	

Valve only	57.2	66.1	

CABG+Valve	12.5	20.1	

CPB time in minutes	104 (53)	146 (63)	< 0.001

Aortic cross clamp time in minutes	73.7 (39.0)	98.6 (44.0)	< 0.001

Use of Mannitol in CPB pump (%)	65.8	73.6	0.06

Use of Albumin in CPB pump (%)	6.4	13.2	0.003

Nadir hematocrit concentration	23.9 (4.4)	22.1 (4.3)	< 0.001

Administration of antifibrinolytic agents (%)			< 0.001

None	39.4	37.9	

Aprotinin	10.4	32.8	

Tranexamic or Aminocaproic acid	50.1	29.3	

Packed red blood cells transfusion	1.11 (1.56)	3.05 (2.93)	< 0.001

Continuous variables are shown as mean (SD) and compared via Student's t-test. Categorical variables are shown as percentages and compared using Chi-squared test. CABG: Coronary artery bypass grafting; CPB: Cardiopulmonary bypass.

**Table 3:** Outcomes according to need of postoperative RRT.

	No postoperative RRT (n = 690)	Postoperative RRT (n = 174)	P value
Days under mechanical ventilation	0.85 (2.37)	8.70 (14.4)	< 0.001

Percent change in creatinine concentration at hospital discharge from preoperative levels.	-0.19 (34.8)	56.8 (152.4)	< 0.001

In-hospital mortality (%)	3.5	65.5	< 0.001

Length of hospital stay in days	14.2 (11.9)	30.2 (31.1)	< 0.001

Continuous variables are shown as mean (SD) and compared via Student's t-test. Categorical variables are shown as percentages and compared using Chi-squared test.

The mean age of the patients was 67 ± 11 years with 60% being men and 99% Caucasians. In-hospital mortality was significantly higher in the RRT group (65.5% versus 3.5%). Length of ICU (20 days versus 4.6 days) and hospital stay (30 days versus 14 days) were also significantly longer in this group. Lower glomerular filtration rates, less basal hemoglobin, diuretic treatment two days prior to surgery, and diabetes were associated with acute kidney injury requiring RRT. Also, we found association between several but not all of the items from the Euroscore [15] and RRT, such as chronic obstructive pulmonary disease (COPD), neurological dysfunction, congestive heart failure, left ventricular ejection fraction, prior cardiac surgery, active endocarditis, critical state, urgent surgery, pulmonary hypertension, and surgery type. As a result, the logistic Euroscore [16] was significantly higher in the RRT group. Patients requiring RRT were more likely to receive albumin during cardiopulmonary bypass (CPB) and aprotinin during the surgery. On the contrary, half of the patients who did not need RRT received other antifibrinolytic agents. Overall, CPB and aortic cross clamp times were longer in those patients who need postoperative RRT. As far as blood products requirements were concerned, patients in the RRT group were more likely to receive more packed red blood cells (PRBCs) units during the procedure or within 48 hours after the operation.

In the multivariate analysis we found the following variables as independent determinants of postoperative acute renal failure requiring RRT (Table 4): lower glomerular filtration rates, left ventricular ejection fraction or basal hemoglobin levels, diabetes, diuretic treatment two days prior to surgery, urgent surgery, aortic cross clamp duration, administration of aprotinin, and number of intraoperative PRBCs units transfused. After conducting an analysis based on a best case matched propensity score with 137 pairs of patients, the variables that remain statistically significant were prior diuretic treatment (odds ratio = 2.05; 95% confidence interval 1.06-3.96), aortic cross clamp duration (odds ratio = 1.02; 95% confidence interval 1.01-1.03), and intraoperative PRBCs units transfused (odds ratio = 1.22; 95% confidence interval 1.04-1.43).

**Table 4:** Multivariate analysis of the predictors for postoperative renal replacement therapy.

Variable	Odds Ratio* (95% confidence intervals)	P Value
**Preoperative**		

COPD	2.54 (1.39-4.61)	0.002

Diabetes	1.74 (1.09-2.78)	0.02

Previous diuretic treatment	1.93 (1.14-3.27)	0.015

Left ventricular ejection fraction (increase in one percent)	0.97 (0.95-0.99)	0.013

Basal Hemoglobin g/dL	0.86 (0.75-0.99)	0.043

MDRD GFR per mL/min/1.73 m^2^	0.97 (0.96-0.98)	< 0.001

Neurological dysfunction	2.20 (0.70-6.97)	0.18

NYHA class 4	1.17 (0.56-2.42)	0.74

Congestive heart failure	1.19 (0.72-1.96)	0.5

Cardiogenic shock	1.33 (0.32-5.51)	0.69

Infectious endocarditis	1.56 (0.47-5.24)	0.47

Pulmonary hypertension	1.80 (0.91-3.56)	0.1

Preoperative IABP	0.94 (0.21-4.11)	0.93

Critical state	1.98 (0.54-7.20)	0.30

Euroscore (for every point)	0.93 (0.75-1.13)	0.45

Prior cardiac surgery	1.47 (0.59-3.58)	0.43

**Intraoperative**		

Urgent surgery	3.13 (1.09-9.01)	0.03

Aortic cross clamp duration (every minute)	1.01 (1.01-1.02)	0.002

Surgery type		

CABG only (reference)		

Valve only	1.56 (0.78-3.09)	0.21

CABG+Valve	1.44 (0.63-3.30)	0.40

Use of albumin in CPB pump	1.07 (0.49-2.31)	0.87

Intraoperative nadir hematocrit concentration ≥ 24%	1.59 (0.95-2.65)	0.08

Administration of antifibrinolytic agents		

None (reference)		

Aprotinin	1.95 (1.07-3.55)	0.043

Tranexamic or Aminocaproic acid	0.57 (0.35-0.95)	0.03

Intraoperative PRBC transfusion (per unit)	1.34 (1.18-1.52)	< 0.001

*Adjusted for all the variables shown in the table, plus age, sex, and hospital source.

MDRD: Modification of diet in renal disease; GFR: glomerular filtration rate; COPD: Chronic obstrutive pulmonary disease; PRBC: Packed red blood cells. NYHA: New York heart association functional classification; IABP: Intra-aortic ballon pump; CABG: Coronary artery bypass grafting

The multivariate analysis presented a good calibration (Hosmer-Lemeshow goodness-of-fit statistic, p = 0.16) and a good discrimination (c-statistic = 0.87). The model with the propensity score also presented a good calibration (Hosmer-Lemeshow goodness-of-fit statistic, p = 0.57) and a good discrimination (c-statistic = 0.77).

In this cohort, patients suffering CSA-AKI requiring RRT had increased adjusted odds for in-hospital mortality (odds ratio = 37.6; 95% confidence interval 18.1-78.2), and required longer hospital stays (12.7 more days; 95% confidence interval 8.6-16.7). Finally, it is of interest to point out that the 43 patients who had presented a preoperative GFR > 30 mL/min/1.73 m^2 ^were discharged from the hospital with a GFR below 30 mL/min/1.73 m^2 ^(according to the MDRD).

## Discussion

The aim of this study was to evaluate the association between several risk factors and CSA-AKI requiring RRT. Identifying high-risk patients might help to in planning measures to prevent complications, such as AKI. In the strength of this study is that a clear measurement of kidney injury was used, such as the need for RRT, and that we were able to gather a large group of cases. Notably, this cohort allowed the simultaneous examination of several variables during the surgery process.

To strengthen our findings, we used a propensity score to perform a best case-matched analysis. Many consider propensity scores to produce a better adjustment for baseline differences than simply including potential confounders in the multivariable model [14]. In our study, this procedure resulted in 137 pairs. To calculate the propensity score, we included age, sex, body mass index, hospital source, glomerular filtration rates, and the Euroscore.

This work adds information to prior examples of research by assessing the impact of several risk factors in a cohort of Spanish patients.

The current data concurs with previous reports showing that the incidence of CSA-AKI increases with patient-related risk factors such as previous impaired renal function, diabetes, low left ventricular ejection fraction, or procedure-related risk factors such as need for urgent surgery, length of cross-clamp time, administration of aprotinin, and red blood cells transfusion [17]. Evidence about prior diuretic treatment is scarce, and low hemoglobin levels could be related with red blood cells transfusion, although it may be plausible that some relationship exists as an indicator of poor health status or renal failure.

Although some of these variables were not significantly associated with risk for suffering postoperative AKI in the propensity score analysis, these variables presented much association towards the same tendency that we had found in the multivariate analysis. Taking into account that we were able to analyze only 137 pairs, these results could be explained by a lack of statistical power.

We decided to use the Euroscore [16] to stratify patients according to their predicted outcome as it had proved to perform well [18]. We were not able to find a relationship between the logistic Euroscore and the need for RRT. Although Toumpoulis et al found an association [19], they used a less stringent definition for acute kidney injury than we did.

We did not find any association between nadir hematocrit concentration = 24% on CPB and RRT. We chose a 24% value based on the results of a previous study [20]. Despite extensive research, the clinical community remains uncertain whether hemodilution during CPB is harmful or helpful [21-23]. Both the complexity in the physiology of tissue oxygen delivery and the observational nature of the studies give us pause when considering them. An alternative explanation for our results might be variations in local blood transfusion policies and the variety of CPB techniques, or the effects of unmeasured confounders. This controversial issue warrants further study and randomized trials to determine the optimal hematocrit concentration management strategy.

Patients who presented lower nadir hematocrit concentrations in surgery were more likely to have been transfused (correlation r = 0.42 p < 0.001). Although an optimal triggering for transfusion cannot be suggested, patients from our cohort might have benefited from a more restrictive perioperative transfusion strategy. Blood transfusion has been shown to be associated with organ dysfunction and worse outcomes [24-26]. Not surprisingly we found a risk increase for needing RRT for every red blood cells unit given.

Antifibrinolytic agents can reduce total perioperative blood loss during cardiac surgery, reducing the need for transfusion, although aprotinin significantly increased the risk of acute kidney injury [27]. Our results also suggest considering the perioperative administration of either aminocaproic or tranexamic acid as safer alternatives when required.

The type of surgery did not seem to incur in increased risk in our cohort. From our point of view, the duration of the surgery and, specially, prolonged aortic cross clamp times are a more accurate measure of CSA-AKI because they can dictate the effects of the CPB circuits [28].

Regrettably, the mortality risk found for CSA-AKI requiring RRT has not changed in the last decade [1], despite advances in medical care. The high mortality rates deserve more attention despite the fact that it could be argued that CSA-AKI requiring RRT accounts only for a small percentage of the outcomes. The point is that RRT is just the "tip of the iceberg" of renal failure, which carries an impaired outcome [29] even in its less severe stages.

There are several limitations to this study. First, causal inferences must not be drawn due to the observational nature of this study. Because the presented results display only association, the risk factors might be mere correlates of a sicker health status and it is possible that factors other than severe AKI led to worse outcomes.

The absence of statistical significance for the association between RRT and some traditional preoperative risk factors might be explained by our small study population, lack of power, or for unknown confounders. Due to the variations in clinical practices among different hospitals and countries, and because we studied on-pump CABG and valve surgery thus excluding other types of cardiac surgery, generalization of these results to other populations should be restricted. However, some risk factors have also been observed in other cohorts of patients [17]; therefore, the effect of those factors relative to their association with CSA-AKI should be noted.

We should state that, while the MDRD Study equation has many advantages, this formula has been shown to underestimate the GFR in healthy subjects and patients older than 70 years, as it was derived from younger patients with chronic renal failure [30].

## Conclusion

We have identified several risk factors associated with CSA-AKI requiring RRT after evaluating several preoperative and intraoperative variables in a Spanish cohort. Prevention of AKI in cardiac surgical patients should be reinforced because it causes poor outcomes.

## Key messages

1) Cardiac-surgery associated acute kidney injury requiring RRT is associated with worse outcomes.

2) Modifiable risk factors for acute kidney injury should be taken into account before undertaking cardiac surgery.

3) High-risk patients should be identified in advance and preventive measures should be available.

## Abbreviations

CSA: Cardiac surgery associated; AKI: Acute kidney injury; RRT: Renal replacement therapy; CABG: Coronary artery bypass grafting; GFR: Glomerular filtration rate; MDRD: Modification of diet in renal disease; COPD: Chronic obstructive pulmonary disease; CPB: Cardiopulmonary bypass; PRBC: Packed red blood cells; OR: Odds ratio; CI: Confidence intervals.

## Competing interests

The authors declare that they have no competing interests.

## Authors' contributions

Study concept and design: JRPV, PMR, and MBR. Acquisition of data: PMR, MV. Analysis and interpretation of data: JRPV, MBR. Drafting of the manuscript: JRPV, MBR. Critical revision of the manuscript for important intellectual content: JRPV, PMR, NGF, and MBR. Statistical analysis: JRPV, MBR.

All authors approved the final version.

## Appendix. Definitions of variables

### Preoperative

Basal serum creatinine: last preoperative value in mg/dL.

Basal hemoglobin: last preoperative value in g/dL.

NSAIDs treatment seven days prior to surgery

ACE treatment two days prior to surgery.

Diuretic treatment two days prior to surgery.

Statins treatment two days prior to surgery.

Diabetes mellitus, subdivided into nondiabetic patients, use of oral therapy, or insulin.

Extracardiac arteriopaty (presence of one or more of the following symptoms): intermittent claudication, carotid occlusion or >50% stenosis, previous or future surgery for vascular disease.

Chronic obstructive pulmonary disease: long-term use of bronchodilators or steroids, or obstructive pattern shown in pulmonary function test.

Hypertension: 1) Previous diagnosis of hypertension, 2) or systolic blood pressure (SBP) > 140 and/or diastolic blood pressure (DBP) > 90 based on two measurements in nondiabetic nonchronic renal disease patients, 3) or SBP > 130 o DBP > 80 in diabetic or chronic renal diasease patients, 4) or use of antihypertensive drugs (except for heart failure).

Neurological dysfunction: Neurological disease severely affecting ambulatory or day-to-day functioning.

Congestive heart failure: Chronic dyspnea on exertion, orthopnea, or paroxysmal nocturnal dyspnea previous to surgery.

Left ventricular ejection fraction estimated by echocardiography, or radionuclide left ventriculography or any similar method.

NYHA 4: New York Heart Association Functional Classification Class IV: any physical activity brings on discomfort and symptoms occur at rest.

Cardiogenic shock: circulatory shock for more than 30 minutes and 1) SBP < 80 and/or cardiac index < 1.8 L/min/m^2^, or 2) pharmacologic or intra-aortic balloon pump support to maintain SBP > 80 and/or cardiac index > 1.8 L/min/m^2^.

Infectious endocarditis: Patient still under antibiotic treatment at time of surgery.

Pulmonary hypertension: Pulmonary artery pressure > 60.

Unstable angina: Angina requiring intravenous nitrates until arrival in the operating room.

Recent miocardial infarction, subdivided into: noninfaction, < 3 weeks before operation, or > 3 and < 9 weeks before operation.

Intra-aortic balloon pump (IABP): Arrival of patient in the operating room with IABP.

Critical state (presence of one or more of the following situations): 1) Ventricular tachycardia, fibrillation or aborted sudden death, 2) preoperative cardiac massage, 3) mechanical ventilation before arrival in the operating room, 4) preoperative intravenous ionotropic support, 5) arrival of patient in the operating room with intra-aortic ballon pump, 6) preoperative urine output < 10 ml/h.

Prior cardiac surgery: any previous cardiac surgery requiring opening the pericardium.

Urgent surgery: Operation must be performed before the beginning of the next working day.

### Intraoperative

Nadir hematocrit concentration: Lowest hematocrit concentration measured on cardiopulmonary bypass.

## Pre-publication history

The pre-publication history for this paper can be accessed here:

http://www.biomedcentral.com/1471-2369/10/27/prepub

## References

[B1] Chertow GM Lazarus JM Christiansen CL Cook EF Hammermeister KE Grover F Daley J Preoperative renal risk stratification Circulation 1997 95 4 878 884 9054745 10.1161/01.cir.95.4.878

[B2] Chertow GM Burdick E Honour M Bonventre JV Bates DW Acute kidney injury, mortality, length of stay, and costs in hospitalized patients J Am Soc Nephrol 2005 16 11 3365 3370 10.1681/ASN.2004090740 16177006

[B3] Hoste EA Cruz DN Davenport A Mehta RL Piccinni P Tetta C Viscovo G Ronco C The epidemiology of cardiac surgery-associated acute kidney injury The International journal of artificial organs 2008 31 2 158 165 18311732 10.1177/039139880803100209

[B4] Vaschetto R Groeneveld AB An update on acute kidney injury after cardiac surgery Acta clinica Belgica 2007 2 380 384 18284004 10.1179/acb.2007.085

[B5] Leacche M Winkelmayer WC Paul S Lin J Unic D Rawn JD Cohn LH Byrne JG Predicting survival in patients requiring renal replacement therapy after cardiac surgery The Annals of thoracic surgery 2006 81 4 1385 1392 10.1016/j.athoracsur.2005.10.009 16564277

[B6] Palomba H de Castro I Neto AL Lage S Yu L Acute kidney injury prediction following elective cardiac surgery: AKICS Score Kidney international 2007 72 5 624 631 10.1038/sj.ki.5002419 17622275

[B7] Thakar CV Arrigain S Worley S Yared JP Paganini EP A clinical score to predict acute renal failure after cardiac surgery J Am Soc Nephrol 2005 16 1 162 168 10.1681/ASN.2004040331 15563569

[B8] Wijeysundera DN Karkouti K Dupuis JY Rao V Chan CT Granton JT Beattie WS Derivation and validation of a simplified predictive index for renal replacement therapy after cardiac surgery Jama 2007 297 16 1801 1809 10.1001/jama.297.16.1801 17456822

[B9] Brown JR Cochran RP Leavitt BJ Dacey LJ Ross CS MacKenzie TA Kunzelman KS Kramer RS Hernandez F Jr Helm RE Multivariable prediction of renal insufficiency developing after cardiac surgery Circulation 2007 116 11 Suppl I139 143 17846294 10.1161/CIRCULATIONAHA.106.677070

[B10] Filsoufi F Rahmanian PB Castillo JG Silvay G Carpentier A Adams DH Predictors and early and late outcomes of dialysis-dependent patients in contemporary cardiac surgery Journal of cardiothoracic and vascular anesthesia 2008 22 4 522 529 10.1053/j.jvca.2008.01.015 18662625

[B11] Candela-Toha A Elias-Martin E Abraira V Tenorio MT Parise D de Pablo A Centella T Liano F Predicting acute renal failure after cardiac surgery: external validation of two new clinical scores Clin J Am Soc Nephrol 2008 3 5 1260 1265 10.2215/CJN.00560208 18463173 PMC2518781

[B12] Levey AS Bosch JP Lewis JB Greene T Rogers N Roth D A more accurate method to estimate glomerular filtration rate from serum creatinine: a new prediction equation. Modification of Diet in Renal Disease Study Group Annals of internal medicine 1999 130 6 461 470 10075613 10.7326/0003-4819-130-6-199903160-00002

[B13] D'Agostino RB Jr Propensity score methods for bias reduction in the comparison of a treatment to a non-randomized control group Stat Med 1998 17 19 2265 2281 10.1002/(SICI)1097-0258(19981015)17:19<2265::AID-SIM918>3.0.CO;2-B 9802183

[B14] Katz MH ed Multivariable analysis 2006 second New York: Cambridge University Press

[B15] Nashef SA Roques F Michel P Gauducheau E Lemeshow S Salamon R European system for cardiac operative risk evaluation (EuroSCORE) Eur J Cardiothorac Surg 1999 16 1 9 13 10.1016/S1010-7940(99)00134-7 10456395

[B16] Roques F Michel P Goldstone AR Nashef SA The logistic EuroSCORE European heart journal 2003 24 9 881 882 10.1016/S0195-668X(02)00799-6 12727160

[B17] Rosner MH Okusa MD Acute kidney injury associated with cardiac surgery Clin J Am Soc Nephrol 2006 1 1 19 32 10.2215/CJN.00240605 17699187

[B18] Nilsson J Algotsson L Hoglund P Luhrs C Brandt J Comparison of 19 pre-operative risk stratification models in open-heart surgery European heart journal 2006 27 7 867 874 10.1093/eurheartj/ehi720 16421172

[B19] Toumpoulis IK Anagnostopoulos CE DeRose JJ Swistel DG Does EuroSCORE predict length of stay and specific postoperative complications after coronary artery bypass grafting? International journal of cardiology 2005 105 1 19 25 10.1016/j.ijcard.2004.10.067 15908026

[B20] Karkouti K Beattie WS Wijeysundera DN Rao V Chan C Dattilo KM Djaiani G Ivanov J Karski J David TE Hemodilution during cardiopulmonary bypass is an independent risk factor for acute renal failure in adult cardiac surgery The Journal of thoracic and cardiovascular surgery 2005 129 2 391 400 10.1016/j.jtcvs.2004.06.028 15678051

[B21] Karkouti K Wijeysundera DN Yau TM Callum JL Cheng DC Crowther M Dupuis JY Fremes SE Kent B Laflamme C Acute kidney injury after cardiac surgery: focus on modifiable risk factors Circulation 2009 119 4 495 502 10.1161/CIRCULATIONAHA.108.786913 19153273

[B22] Murphy GJ Reeves BC Rogers CA Rizvi SI Culliford L Angelini GD Increased mortality, postoperative morbidity, and cost after red blood cell transfusion in patients having cardiac surgery Circulation 2007 116 22 2544 2552 10.1161/CIRCULATIONAHA.107.698977 17998460

[B23] Spiess BD Choose one: damned if you do/damned if you don't! Critical care medicine 2005 33 8 1871 1874 10.1097/01.CCM.0000174499.98087.40 16096475

[B24] Spiess BD Transfusion of blood products affects outcome in cardiac surgery Seminars in cardiothoracic and vascular anesthesia 2004 8 4 267 281 10.1177/108925320400800402 15583789

[B25] Slight RD Nzewi O McClelland DB Mankad PS Red cell transfusion in elective cardiac surgery patients: where do we go from here? British journal of anaesthesia 2009 102 3 294 296 10.1093/bja/aep001 19218370

[B26] Koch CG Li L Duncan AI Mihaljevic T Cosgrove DM Loop FD Starr NJ Blackstone EH Morbidity and mortality risk associated with red blood cell and blood-component transfusion in isolated coronary artery bypass grafting Critical care medicine 2006 34 6 1608 1616 10.1097/01.CCM.0000217920.48559.D8 16607235

[B27] Brown JR Birkmeyer NJ O'Connor GT Meta-analysis comparing the effectiveness and adverse outcomes of antifibrinolytic agents in cardiac surgery Circulation 2007 115 22 2801 2813 10.1161/CIRCULATIONAHA.106.671222 17533182

[B28] Verrier ED Boyle EM Jr Endothelial cell injury in cardiovascular surgery The Annals of thoracic surgery 1996 62 3 915 922 10.1016/S0003-4975(96)00528-0 8784042

[B29] Chertow GM Levy EM Hammermeister KE Grover F Daley J Independent association between acute renal failure and mortality following cardiac surgery The American journal of medicine 1998 104 4 343 348 10.1016/S0002-9343(98)00058-8 9576407

[B30] Rule AD Larson TS Bergstralh EJ Slezak JM Jacobsen SJ Cosio FG Using serum creatinine to estimate glomerular filtration rate: accuracy in good health and in chronic kidney disease Annals of internal medicine 2004 141 12 929 937 15611490 10.7326/0003-4819-141-12-200412210-00009

